# Accelerated Weathering of Polylactide-Based Composites Filled with Linseed Cake: The Influence of Time and Oil Content within the Filler

**DOI:** 10.3390/polym11091495

**Published:** 2019-09-12

**Authors:** Olga Mysiukiewicz, Mateusz Barczewski, Katarzyna Skórczewska, Joanna Szulc, Arkadiusz Kloziński

**Affiliations:** 1 Institute of Material Technology, Poznan University of Technology, Piotrowo 3, 61-138 Poznan, Poland; mateusz.barczewski@put.poznan.pl; 2 Faculty of Technology and Chemical Engineering, University of Technology and Life Science, Seminaryjna 3, 85-326 Bydgoszcz, Poland; katarzyna.skorczewska@utp.edu.pl (K.S.); joanna.szulc@utp.edu.pl (J.S.); 3 Institute of Chemical Technology and Engineering, Poznan University of Technology, Berdychowo 4, 60-965 Poznan, Poland; arkadiusz.klozinski@put.poznan.pl

**Keywords:** polylactide, bio-based composites, waste filler, accelerated weathering

## Abstract

This paper presents the effects of accelerated weathering on the properties of polylactide (PLA) composites filled with linseed cake. The particle-shaped waste filler with different linseed oil content (0.9–39.8 wt %) was incorporated with constant amount of 10 wt % to a polymeric matrix and subjected to accelerated weathering tests with different exposition times. The structure of the composites, their mechanical, thermal, and thermo-mechanical properties were evaluated by means of scanning electron microscopy, tensile test, dynamic mechanical thermal analysis, and differential scanning calorimetry prior to and after weathering. The results of the measurements were analyzed in reference to the amount of crude oil contained in the filler. The behavior of the multiphase composite during weathering was described. It was found that the oil-rich samples during the first stage of the process showed increased resistance to hydrolytic degradation due to their relatively high crystallinity. The presence of water and elevated temperatures caused swelling of the filler and cracking of the polymeric matrix. Those discontinuities enabled the plasticizing oil to be rinsed out of the composite and thus water penetrated into the samples. As a result, the PLA-based composites containing oil-rich linseed cake were found to be more vulnerable to hydrolytic degradation in a longer time.

## 1. Introduction

Polymeric materials are lightweight, durable, easy to produce, and cheap—therefore they dominate the market of consumer products. Unfortunately, the production of plastic products characterized with a short lifespan results in excessive amounts of waste, which affects the natural environment. Even though the majority of popular plastics such as polyolefins or polyesters can be recycled, other solutions need to be implemented in order to manage plastic waste and thus effectively protect the environment.

One of the available methods of achieving this goal is replacing conventional polymers with their bio-based and biodegradable counterparts. Various environmentally-friendly plastics have been proposed, including thermoplastic starch, polyhydroxyalkanoates (PHA) and polylactide (PLA) [1,2,3,4,5,6,7]. PLA is commonly used due to its good mechanical properties and possible conventional processing technology applications [8]. What is more, the properties of this polymer can be tuned for specific applications using different modifying agents such as plasticizers, nucleating agents or chain extenders [9,10]. Polylactide can also be successfully used as a matrix for polymeric composites [11,12].

Even though PLA is mostly used for short-lifespan applications such as food packages or drinking cups, it is becoming increasingly popular in the automotive, electronic or fiber industries where reliability is prioritized [13]. Therefore, the knowledge of ageing behavior is crucial to fully benefit from the polylactide’s utilization. It is common knowledge that this biodegradable polymer is susceptible to both enzymatic and hydrolytic degradation. During the latter process, water diffuses into the polymeric matrix and causes a cleavage reaction of the polymeric chains. The polar, water-soluble low molecular weight products of the hydrolysis further promote the degradation of PLA due to the presence of carboxylic end groups [13,14]. There are numerous factors which influence the process of hydrolytic degradation of PLA. External factors such as temperature or the aqueous medium’s pH can be distinguished from the internal ones, including the polymer’s crystallinity [13,14] or the presence of modifying agents. It is generally accepted that the first stage of hydrolysis takes place in the amorphous regions of the polymer, where the diffusion of water is possible. Degradation of the crystalline domains can be observed in the second stage of this process [14]. Hydrolysis of the PLA matrix also changes due to the addition of various modifying agents and fillers [15]. In the case of the polylactide-based composites, the interface between the filler and the polymer is crucial for the hydrolysis. When the affinity between the filler and the polymer is insufficient, due to the presence of gaps and discontinuities, water can penetrate the composite more easily, thus facilitating the degradation. The application of coupling agents or chemical treatment of the filler also influences hydrolytic degradation of the composite. Gill-Castel et al. found that the application of maleic anhydrite improves the adhesion of sisal fibers and PLA matrix making the composites more resilient to degradation [16]. As described by Islam, Pickering, and Foreman in the case of alkali treatment of hemp fiber, the polylactide composites also caused showed resistance to ageing in humid conditions [17]. What is more, some of the natural fillers contain active ingredients which inhibit degradation of the composite [18].

The so-called waste fillers are gaining popularity among the environmentally-friendly fillers with potential modifying ability [19]. Waste fillers are various additives to composites, which are by-products from different branches of industry. Agriculture or food industry-derived plant-based waste fillers are especially advantageous as they are biodegradable and CO_2_-neutral. What is more, they mostly comprise of lignocellulose, therefore they can replace wood flour or natural fibers. Additionally, plant-based waste fillers oftentimes contain active ingredients such as essential oils or tannins, which may provide a modifying effect. Different waste fillers such as nut shells [20,21], grain husks [22,23], seeds [24,25], fruit waste [26] or even fish scales [27] have been proposed for polymeric composites. Linseed cake (LC), a by-product of mechanical oil extraction from linseed (*Linum usitatissimum* L.), is an advantageous filler for PLA-based composites [28,29]. It contains up to 30 wt % of vegetable oil, which increases the segmental mobility of macromolecules. This behavior simultaneously causes a plasticizing effect and increases polylactide crystallinity. As a result, LC-filled composites are characterized with high crystallinity, good deformability, and low viscosity. Unfortunately, linseed oil (which provides the modifying effect) is not fully miscible with the polymeric matrix [30]. As both the filler and the polymer are biodegradable, the influence of the LC and the oil on the properties of the composites may change over time. What is more, Mittal et al. studied PLA-based composites filled with date seed powder and showed that the vegetable oil migrates from the filler during its lifespan [24]. Therefore, it may be hypothesized that the efficiency of the composite’s plasticization can be influenced during the ageing of this product. Moreover, the addition of linseed cake may potentially increase the resistance to hydrolysis of the composites by increasing the polymeric matrix’s crystallinity.

The aim of the study is to analyze the influence of linseed oil on the weathering behavior of polylactide-based composites.

## 2. Materials and Methods

### 2.1. Materials

A commercially available grade of polylactide Ingeo 2500 HP by Nature Works (Minnetonka, MN, USA, melt flow rate of 8 g/10 min _(190 °C, 2.16 kg)_ and the density of 1.24 g/cm^3^) was used as the matrix of the composites.

**Figure 1:**
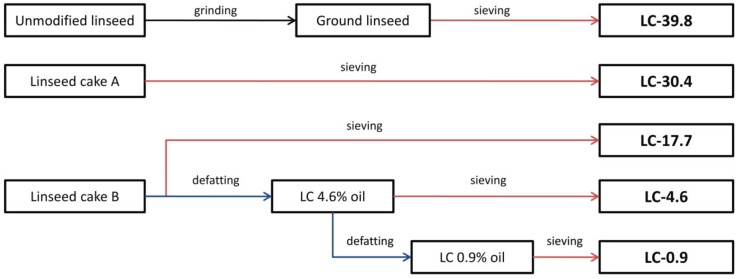
Schematic representation of the filler preparation.

Two grades of linseed cake with different oil content (LCA and LCB) as well as unmodified linseed were obtained from a local Polish supplier. In order to further diversify the oil content in the fillers, LCA was divided into three parts. One part was left unmodified, one was subjected to oil removal using the solvent method, according to the procedure described in our previous work (mechanical stirring with acetone, t = 30 min, r = 300 rpm) [28], whereas for one part the oil removal procedure was repeated twice. Unmodified linseed was subjected to grinding using a Bosch MKM 6003 high speed knife grinder (Gerlinger, Germany). All of the used fillers were sieved with a Fritsch Analysette Pro 3 sieve shaker (Idar-Oberstein, Germany) equipped with a 630 µm mesh. A schematic representation of the preliminary filler preparation procedure with the LC series assignment dependent on oil content which was used in the study is shown in Figure 1.

The standard Soxhlet extraction procedure was employed in order to determine total oil content in the unmodified and as-received fillers. A Büchi Universal Extraction System B-811 (Flawil, Switzerland) was used, with petroleum ether as a solvent and a total extraction time of 150 min. The crude oil content values in LCA defatted twice, LCA defatted once, LCA, LCB, and ground linseed were 0.9, 4.6, 17.7, 30.4 and 39.8 wt %, respectively.

**Figure 2:**
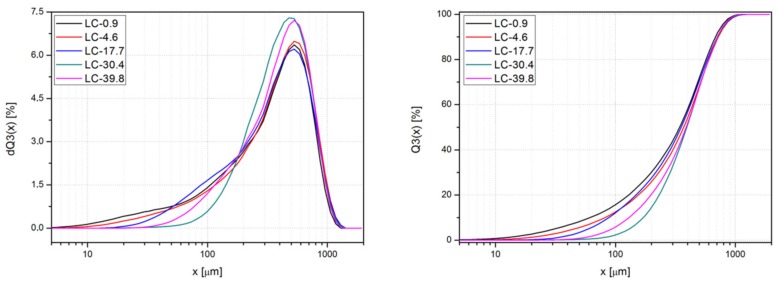
The particle size distribution curves Q3(x) and their derivatives dQ3(x) as a function of the particle size x obtained for the fillers.

The particle size of different grades of linseed cake was evaluated with a laser particle sizer Fritsch Analysette 22. The particle size distribution curves Q3(x) and their derivatives dQ3(x) obtained for the fillers are presented in Figure 2 as a function of the particle size x. The linseed cake particles, regardless of the oil content or the preparation method, are characterized by a size within the range of 5–1100 µm, with the mean value of 480 µm in the case of LC-39.8 and 530 µm for the remaining grades. It can also be noticed that linseed cake with a lower amount of crude oil (17.7 wt % and less) contained more particles smaller than 200 µm compared to its LC-30.4 and LC-39.8 counterparts. It may be concluded that the crude oil on the surface of the waste filler particles promotes the formation of aggregates, hence the lack of the small particle fraction in the case of the linseed cake characterized with a higher oil content. However, it should be observed that the preliminary treatment of the filler does not markedly influence the particle size nor the Q3(x) curve shape. Therefore, it may be concluded that any changes in the composites filled with different grades of linseed cake are caused by the differences in the fillers’ composition rather than their particle size.

### 2.2. Sample Preparation

The polymeric composites containing 10 wt % of the filler were manufactured using polylactide as the matrix. The choice of 10 wt % content of the filler was based on our previous studies, since beneficial modifying effects were observed for this LC concentration [28]. Both the filler and the polymer were physically mixed and dried at 70 °C overnight in a Memmert ULE500 laboratory cabinet drier (Schwabach, Germany) prior to processing. The mixing of the components in a molten state was performed, using a ZAMAK EH-16.2 D co-rotating twin screw extruder (Skawina, Poland) operating at 120 rpm and 190 °C. The extrudates were cooled at air temperature, pelletized, and dried as before. Samples were injection molded using a Battenfeld PLUS-35 machine (Kottingbrunn, Austria) with the following parameters: T_injection_ = 210 °C, T_mold_ = 50 °C, p_injection_ =72 MPa, v_injection_ = 75 mm/min for further testing. The samples were named in reference to the type of the used filler, e.g., PLA-LC-39.8 is the composite sample containing 10 wt % of the filler containing 39.8 wt % of crude oil.

The accelerated weathering process was conducted using a QLAB QUV chamber (Westlake, OH, USA) according to the ISO 4892-3 standard for 250 and 500 h. The samples were exposed to UV light (340 nm, 0.76 W/m^2^) as well as periodically sprayed with water—an 18 min spraying was followed by a 102 min dry period. The temperature inside the chamber was set to 60 °C, which is slightly below the glass transition temperature of polylactide. The weathered samples were conditioned in room conditions for at least seven days before testing.

### 2.3. Methods

The density of the composite samples was determined using the hydrostatic method with distilled water as the immersion fluid (ρ_water,23 °C_ = 0.9975 g/cm^3^). The sample mass was measured in the air (m_1_) and then after submerged in water (m_2_) using an AXIS AD200 balance (Gdańsk, Poland). The density of the material was calculated according to the following formula (1):

(1) $$\rho =\frac{m_{1}\cdot \rho_{water,\;23{}^{\circ}C}}{\left(m_{1}-m_{2}\right)}$$

The density of the waste fillers was measured with a Helium pycnometer Thermo Scientific Pycnomatic (Waltham, MA, USA) according to the ASTM 792-66 standard. 

The surface of the selected samples was examined using a Leevenhuk optical microscope (Tampa, FL, USA) equipped with a 4× lens. The pictures of the unaged and weathered samples were digitally captured.

Surfaces of the composites’ brittle fractures were subjected to scanning electron microscope (SEM) observations using a Vega Tescan apparatus (Brno, Czech Republic). Prior to the tests, the examined surfaces were sputtered with carbon. The electron accelerating voltage of 12.0 kV was applied. The magnification of 3000× was applied. The images were digitally captured.

Differential scanning calorimetry (DSC) was applied in order to evaluate the thermal properties of the composite samples and pure PLA, as well as their crystallinity. The samples of approximately 5.0 ± 0.2 mg were placed in standard aluminum crucibles with pierced lids. The measurement was performed using a Netzsch DSC 204F1 Phoenix apparatus (Selb, Germany). The material was heated from 20 °C to 190 °C, held in a molten state for 10 min and cooled back to the initial temperature, with a heating/cooling rate of 10 °C/min and an inert Nitrogen atmosphere. This cycle was repeated twice in order to evaluate the properties of the composites independently from their thermal history. The crystallinity degree X_C_ was calculated according to the following formula (2)

(2) $$X_{C}=\frac{\Delta H_{M}-\Delta H_{CC}}{\left(1-\varphi \right)\Delta H_{100\%PLA}}\cdot 100\%$$

where: Δ*H*_M_—is the melting enthalpy of a sample, Δ*H*_CC_—is the cold crystallization enthalpy of a sample, Δ*H*_100%PLA_—is the melting enthalpy of the 100% crystalline PLA, Δ*H*_100%PLA_ = 93 J/g [31], φ—is the filler content, and φ = 0.1 for the composite samples.

In order to evaluate the thermo-mechanical properties of the investigated samples, the dynamic mechanical thermal analysis (DMTA) was implemented, using an Anton Paar MCR 301 apparatus (Graz, Austria). The measurements were conducted on 50 × 10 × 4 mm^3^ rectangular samples, in the torsion mode, at a frequency of 1 Hz and strain of 0.01%. The temperature range was set to 30–110 °C, with the heating rate of 2 °C/min. Storage modulus *G*′, loss modulus *G*″, and loss factor tanδ values were determined as a function of the temperature. The position of tanδ maximum was used to determine the glass transition temperature *T*_g_ of the neat polymer and its composites.

Mechanical properties in static conditions, such as tensile strength Rm, tensile modulus *E*, and elongation at break ε of the samples were evaluated by means of the static tensile test, according to the ISO 527-2 standard. A Zwick Z010NT universal testing machine (Ulm, Germany) was used. The crosshead speed was 1 mm/min during the determination of the tensile modulus and 50 mm/min during the remaining part of the test.

The impact strength of PLA and PLA-based composites was assessed according to the DIN 53435 standard using a 0.98 J hammer and Dys-e 8421 apparatus. Unnotched samples of 15 × 10 × 4 mm^3^ were tested.

## 3. Results and Discussion

### 3.1. Density

The evaluation of the volume void content of a composite material helps to assess the quality of the material, especially the adhesion between the filler and the matrix. As it is based on the comparison of theoretical and measured density of the sample, it provides useful information on the whole volume of the tested material, not just its fracture surface (as in the case of microscopic observations). The theoretical density ρ_T_ of the composite materials was calculated according to the following formula (3):

(3) $$\rho_{T}=\frac{\rho_{M}\cdot \rho_{F}}{W_{M}\cdot \rho_{F}+W_{F}\cdot \rho_{M}}$$

where: *ρ_M_*—density of the composite matrix, *ρ_F_*—density of the filler, *W_M_*—weight content of the matrix, *W_F_*—weight content of the filler.

The void volume content *u_V_* of the samples was calculated according to the following formula (4) [32]:

(4) $$u_{V}=\frac{\rho_{T}-\rho_{R}}{\rho_{T}}\cdot 100\%$$

where *ρ_R_*—is the measured density of the composite matrix.

The values of measured density, theoretical density, and volume void content of the fillers, neat PLA, and composite samples subjected to accelerated ageing are collected in Table 1. The *u_V_* could not be calculated for the weathered samples, as the density of the fillers subjected to ageing could not be measured.

The void volume content of all the composite samples did not exceed 2%, which indicates good quality and lack of porosities [31,33]. The lowest value was denoted for the PLA-LC-4.6 sample and the highest for the PLA-LC-39.8 one. A considerably high value of 0.94% was also found for the composite containing the lowest amount of linseed oil. As epoxidized vegetable oil acts as a compatibilizing agent for PLA-based lignocellulose-filled composites [30], presumably its presence helped to achieve good affinity between the phases of the samples, hence the lack of porosities. However, when an excess of crude oil is present in the composite, it is no longer miscible and it creates a separate phase in the form of oil-filled voids [34], as in the case of the PLA-LC-39.8 sample. The volume void content value calculated for the neat resin can be ignored as it shows the differences between the manufacturer’s data and the actual density value. 

The density of the studied composites depended on both their composition and weathering. It can be observed that the density of the LC-0.9 filler is almost equal to the one of typical flax fiber [35] and decreases along with the growing content of low-density crude oil, characterized with a specific gravity of 0.925 [36]. A reduction in the material’s density and consequently in the weight of a part resulting from the addition of linseed cake is an advantageous outcome, especially for manufacturing purposes. The density of the neat polymer as well as the composite specimens changed due to accelerated weathering. Even though an increase in weight of the PLA-based samples’ weathered for less than 750 h is reported in the literature [17], in the case of the linseed-cake filled specimens an opposite effect can be noticed. The only exception is the PLA-LC-39.8 sample weathered for 250 h whose density slightly increased. The increase of the samples’ density is usually explained by the absorption of water, whereas in this study the composites were conditioned in room conditions prior to testing, which allowed the absorbed moisture to evaporate. A decrease in the density of PLA-based samples may be attributed to the formation of microcavities and voids [37] due to swelling of the lignocellulosic filler and leaching the low molecular fractions out of the polymer during ageing [17]. 

### 3.2. Morphology

**Figure 3:**
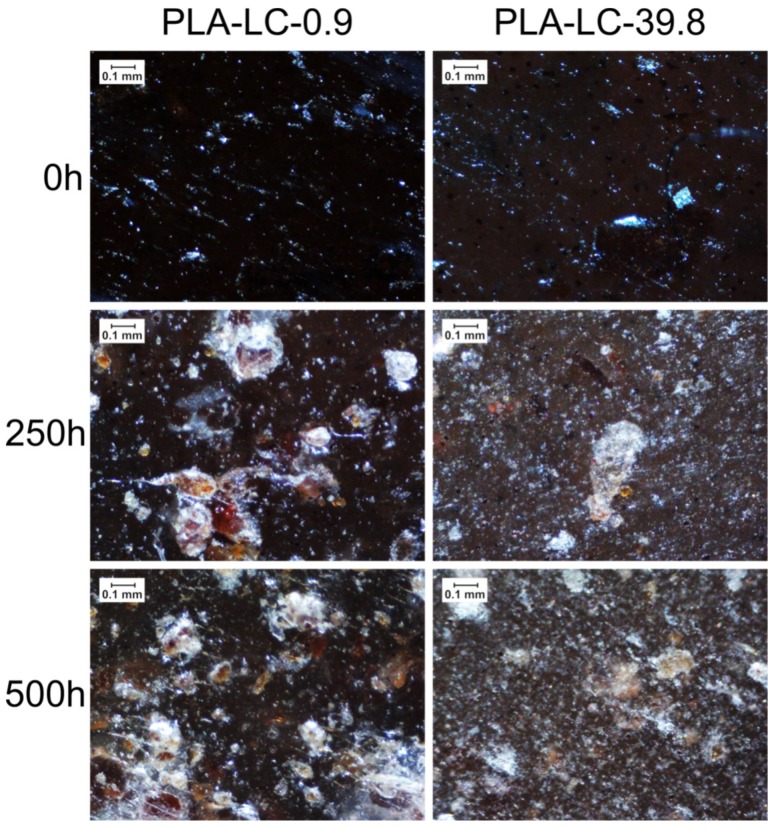
Surfaces of polylactide-linseed cake (PLA-LC)-0.9 and PLA-LC-39.8 samples weathered for different periods.

The images of the samples’ surfaces are shown in Figure 3. In order to keep the discussion simple, only the appearance of PLA-LC-0.9 and PLA-LC-39.8 weathered for different periods are presented for comparison.

As can be observed, the surface of the unaged samples is dark and smooth, with several filler particles dispersed homogenously. They are easier to notice in the case of the PLA-LC-39.8 sample, which is lighter. The difference in the appearance of the two specimens may be caused by differences in crystallinity and the resulting optical properties (to be discussed in detail in paragraph 3.3). 

Accelerated weathering for 250 h resulted in a prominent change in the samples’ appearance, regardless of the oil content in the filler. The surfaces of the composites are noticeably lighter, but the most significant change can be seen in the case of the filler particles. They are easily visible in the whole studied area because of light discoloration around them. This effect can be attributed to small cracks in the polymeric matrix surrounding the filler, caused by its cyclic dilatation during weathering [17,38]. Usually, when the PLA is subjected to elevated temperatures and humidity, changes in its structure occur, resulting in shrinkage as well as embrittlement [39]. On the other hand, the lignocellulosic filler particles swell, thus causing an increase in stress on the interface and subsequent cracking of the composites. Apart from the filler particles, different discontinuities such as voids and porosities can also contribute to the deformation-caused cracking. On the other hand LC, as a polysaccharide-rich organic waste filler, is a highly hygroscopic substance [40,41], which increases its volume due to the absorption of water. As the polymeric matrix does not show this behavior, the filler particles stretch it. Due to the insufficient ability to deform the PLA matrix, cracks occur. What is more, surface whitening caused by changes in the refraction index was observed in the case of hydrolytic degradation of PLA, due to the presence of hydrolysis products and absorbed water [42]. Interestingly, surface whitening did not noticeably intensify due to weathering for 500 h.

**Figure 4:**
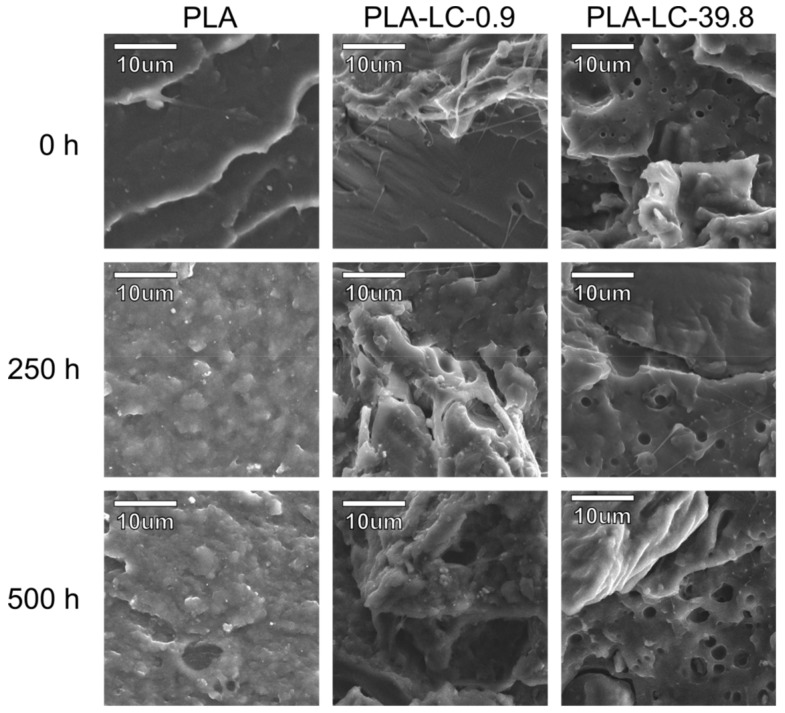
SEM images of the chosen samples weathered for 0, 250, and 500 h.

Images of the samples’ brittle fractured surfaces obtained by scanning electron microscopy are presented in Figure 4. The sample of non-weathered, unmodified polylactide reveals a smooth fracture surface typical for the amorphous form of this polymer. The incorporation of 10 wt % of defatted linseed cake (LC-0.9) did not considerably change the character of the fracture. The lignocellulosic particles are partially covered with the polymeric matrix, therefore a good affinity between the phases can be noticed. In the case of the addition of the oil-rich filler (LC-39.8), the morphology of the composites changed. Small (<1 μm) spherical domains, which can be seen dispersed on the fracture surfaces can be identified as oil droplets [25]. Linseed oil is only partially miscible with polylactide, therefore its excess oozes as separate domains. Similar behavior was observed in the case of PLA modified with epoxidized linseed or soybean oil [30,43].

Accelerated weathering of polylactide results in changes in its microstructure. The sample weathered for 250 h shows a typical morphology for semi-crystalline polymers deformed in a plastic way. This behavior may be explained by the degradation of the amorphous phase and solid-state recrystallization due to the presence of water and elevated temperature. The pure PLA sample subjected to accelerated weathering for 500 h can also be characterized with a fracture surface typical for the crystalline form, however, it is more uneven compared to the one weathered for 250 h. This change may suggest the degradation of the crystalline as well as amorphous regions. 

The composites filled with the LC-0.9 filler subjected to weathering for 250 h show a fracture surface typical to semi-crystalline polymers, similarly to the neat polymer. The appearance of the polymeric matrix is not the only change due to weathering—in this case cracks or gaps can be observed on the interface, which is in good agreement with the results of surface observations. Weathering for 500 h further reduced the affinity between the filler and the matrix—in this case the LC particles cannot be seen on the fracture surfaces, as they were pulled out of it during the impact break of the sample.

The morphology of the PLA-LC-39.8 samples subjected to the accelerated weathering changed in three different ways. First of all, the appearance of the polymeric matrix is typical to the semi-crystalline polymer, as in the case of the neat PLA. Cracks are also visible on the interface, as in the case of the PLA-LC-0.9 composites. Unlike in the remaining cases, the oil droplets become noticeably bigger. It is especially noticeable for the sample weathered for 500 h—in this case the diameter of the oil domains exceeds 5 μm, and they are also connected with each other, creating a network throughout the composites’ volume. Presumably, oil leaches out of the composite during ageing. The former droplets become voids which can be filled with water. Therefore, the hydrolysis of the polymer can begin within these domains. The degradation products can be easily removed (i.e., be rinsing out) and the hydrolysis proceeds, thus resulting in the increase in the domains’ dimensions.

### 3.3. Thermal Properties

**Figure 5:**
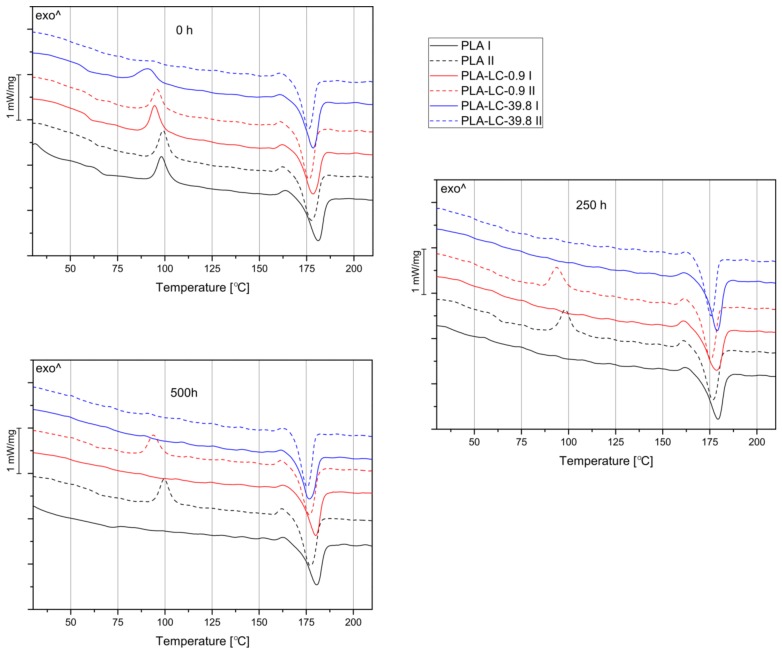
Differential scanning calorimetry (DSC) curves obtained during the first (I) and second heating (II) of selected samples weathered for 0, 250, and 500 h.

The crystallinity values of PLA and PLA-based composites calculated according to formula (2) are collected in Table 2 and selected DSC thermograms are presented in Figure 5. When comparing X_C_ values of unaged samples, a positive correlation between the crystallinity and linseed oil content can be observed. The crystallinity of the PLA-LC-39.8 sample measured during the second heating is over twice as high as in the case of the pure PLA. This behavior is in line with what we found in our previous studies, i.e., that the presence of linseed oil makes the crystallization of the polymer easier by increasing the chain mobility [28]. Similar results have been obtained in the case of the PLA modified with different plasticizing agents, such as polyethylene glycol [44], epoxidized fatty acid esters [45] or epoxidized linseed oil [30]. What is more, the X_C_ value of unaged samples calculated during the first heating was considerably lower in comparison to the results obtained during the second heating. Apparently, the low mold temperature during injection molding resulted in a high cooling rate of the molten polymer, therefore suitable conditions for effective melt crystallization of PLA were not achieved. 

Both composite and unfilled samples subjected to accelerated weathering for 250 h reveal higher crystallinity calculated during the first heating in comparison to the reference samples. Similar results have already been reported in the case of weathered PLA-based composites [17,46]. As the temperature during accelerated weathering was only 60 °C, this rearrangement of the crystalline structure was caused by the simultaneous influence of both heat and UV radiation, inducing partial degradation of the polymer and the process known as chemi-crystallization [17,47]. The growth of X_C_ values measured by DSC is an effect of macromolecular rearrangement of the PLA amorphous phase as a result of chain scission mechanism caused by the initial degradation of PLA, starting in the amorphous regions [14]. The greatest difference of crystallinity can be observed for the PLA-LC-0.9 sample, whose X_C_ increased from 27.01% to 71.55%. The magnitude of changes decreases with the growing vegetable oil content, the smallest growth was denoted in the case of the PLA-LC-39.8 composite. The samples already characterized by high crystallinity did not show such profound changes because both the solid state recrystallization and hydrolytic degradation predominantly take place in the amorphous regions [14,48].

When comparing the changes of samples’ crystallinity measured during the first and second heating, it is possible to divide the temporary effects of chemi-crystallization from permanent changes caused by accelerated ageing. X_C_ values of the samples weathered for 250 h are higher than the ones of the reference specimens, but the increase is not as pronounced as in the case of the first heating—the growth did not exceed 28% for the neat resin and 21% for the composite samples. During the degradation of polylactide the chain scission leads to a decrease in the polymer’s molecular weight. The shorter macromolecules are able to create the crystalline phase more easily, hence the increase in X_C_ values [47,49]. Therefore, an increase in the samples’ crystallinity indirectly indicates a decrease in the molecular weight due to accelerated weathering. The composite samples characterized with high X_C_ value before ageing, such as PLA-LC-30.4 and PLA-LC-39.8 show a less pronounced increase in crystallinity compared to the remaining specimens. Once again, the reason is their lower initial content of the amorphous phase, which makes them less susceptible to degradation [14]—hence smaller changes in their structure. 

The crystallinity of the samples weathered for 500 h calculated during the first heating is higher in comparison to the unaged specimens, but lower in reference to the composites weathered for 250 h. This result indicates that the second stage of the hydrolytic degradation, in which the crystalline as well as amorphous domains are subjected to hydrolysis, occurred [14]. When comparing the crystallinity of the samples calculated during the second run of DSC, a growth can be noticed in the case of PLA and PLA-LC-0.9 only. Once again, this behavior can be explained by polymeric chain scission and the resulting higher mobility of macromolecules [47]. The composites containing a higher percentage of vegetable oil show a decrease in X_C_ values. It may be hypothesized that during the accelerated weathering linseed oil is rinsed out of its domains, which can be then filled with water. In this way, hydrolytic degradation can occur not only on the surface of the composites, but throughout their entire volume. Another explanation of the suppression of the crystallization process is the presence of molecular defects caused by hydrolysis and UV radiation of the polymer [47].

During the first stage of degradation, vegetable oil-rich samples are more stable due to fewer amorphous domains. When the crystalline domains are also subjected to degradation, the non-homogeneous structure of the oil-rich samples become more vulnerable to water, which can penetrate the whole volume of the composite, thus accelerating its degradation.

### 3.4. Thermomechanical Properties

**Figure 6:**
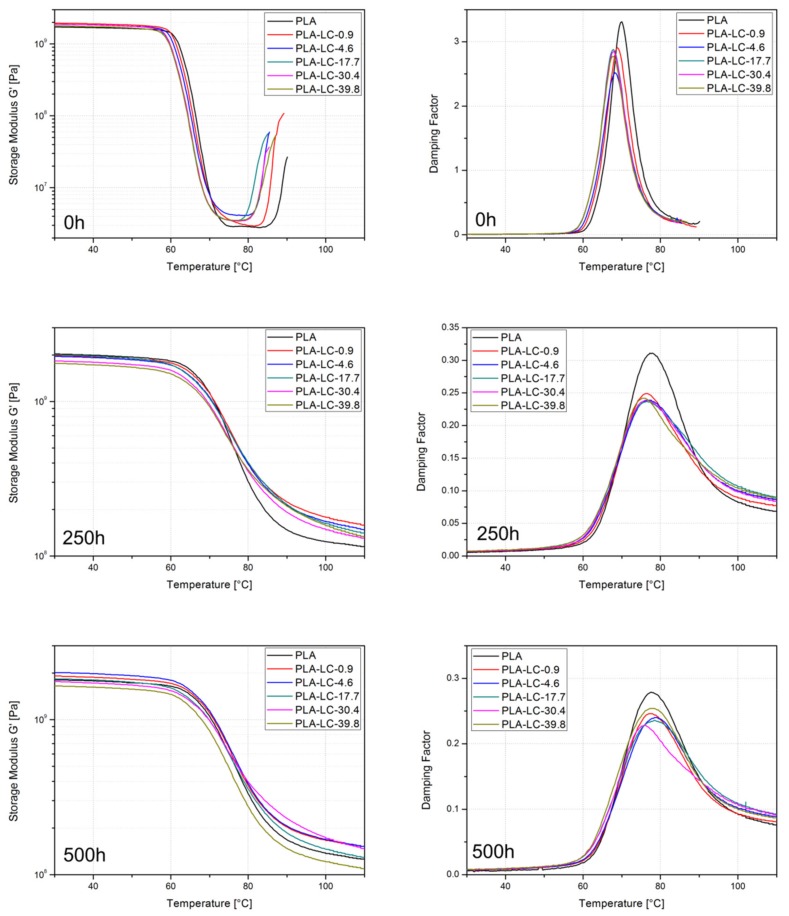
The run of storage modulus (*G*′) versus temperature, loss modulus (*G*″) versus temperature and damping factor (tanδ) versus temperature curves for the composite samples weathered for different periods of time.

The results of dynamic mechanical thermal analysis, i.e., the values of storage modulus *G*′, loss modulus *G*″, and damping factor tanδ as functions of the temperature are presented in Figure 6. A typical run of *G*′ compared to the temperature curve observed for amorphous polylactide consists of four stages: An initial plateau in the glassy state, a decrease attributed to glass transition followed by a plateau in the rubbery state, and an increase due to cold crystallization. These four stages can be easily observed for the untreated samples, regardless of their composition. Even though the shape of *G*′ as a function of the temperature does not change and the storage modulus of the composite samples in the glassy state does not differ notably, some differences between the samples can be seen. First of all, the specimens containing linseed cake enter the second stage (i.e., the decrease of *G*′) at lower temperatures. The increase of the storage modulus attributed to crystallization also begins earlier for the LC-filled composites. This observation is consistent with the results of the DSC measurements, which shows that the incorporation of oil-rich waste fillers causes a decrease of the cold crystallization temperature. The fact that the composite samples enter the phase transition at lower temperatures in comparison to the neat polymer may also indicate that the presence of linseed oil is characterized by low molecular weight and relatively low viscosity, which promotes the movements of macromolecules.

The shape of *G*′ curve changes due to accelerated weathering for 250 and 500 h. In this case, the transition between the glassy and rubbery state is not as sharp and the observed decrease of the *G*′ value is much smaller. What is more, the cold crystallization no longer occurs. Increased crystallinity of the samples (also confirmed by the DSC measurements and SEM observations) is the reason for this behavior—the crystalline domains retain their storage modulus when the amorphous part of the polymer gains increased mobility and enters the rubbery region. Interestingly, the composite samples weathered for 250 h show higher storage modulus values at elevated temperatures in comparison to the neat polymer. This difference may be explained by two phenomena: Increased crystallinity of the LC-filled samples, or the stiffening influence of the rigid lignocellulosic particles within the linseed cake. In the case of the untreated samples, this influence may have been suppressed by the presence of the plasticizing oil. As the oil leaches during weathering, the reinforcing effect of LC dominates, causing an increase in the *G*′ values. Nevertheless, this correlation does not fully apply to the composites weathered for 500 h—as the hydrolysis of the crystalline regions can be observed in their case, the increased crystallinity no longer provides the stiffening effect. What is more, as the SEM observations showed, the oil-rich samples due to their non-homogeneous morphology are subjected to a more intensive degradation within their whole volume, so the reinforcing effect of lignocellulosic is limited.

When analyzing the shape of the damping factor as a function of the temperature, a single peak can be observed for all the samples, both the newly made and the weathered ones. This maximum can be attributed to glass transition, i.e., relaxation of the amorphous regions. A very sharp peak can be seen for the unaged samples. It is slightly higher for the neat polymer in comparison to the composites. This can be explained with higher crystallinity and the presence of rigid structures in the LC-filled composites. However, the amount of linseed oil does not visibly influence the damping properties of the samples. The specimens subjected to accelerated weathering show a much lower and less defined, as well as wider tanδ peak. This change can be explained by different processes which take place in the polymer’s structure due to weathering. First of all, their crystallinity increases, the material becomes stiffer and its damping ability is lower—hence the decrease in the peak’s height. What is more, the hydrolysis of the polymer along with the shortening of macromolecules lead to higher polydispersity. The presence of polymeric chains characterized by different molecular weights and different lengths results in a wider temperature range. 

The values of glass transition temperatures understood as the tanδ maximum for the studied samples are presented in Table 3. The *T*_g_ value for pure, untreated PLA is 70.2 °C. The glass transition temperature decreases along with the increasing linseed oil content in the filler to 67.4 °C measured for the PLA-LC-38.9 sample. Even though the change is rather small, the overall observable trend indicates that the oil-rich filler has a plasticizing effect on the polylactide-based composites. Similar behavior was observed in the case of the PLA plasticized with different vegetable oils, including epoxidized linseed oil [50].

The samples weathered for 250 h are characterized by higher *T*_g_ values in comparison to the untreated ones. The highest value of 77.3 °C was observed for the neat polymer and the lowest temperature of 75.2 °C was denoted in the case of the PLA-LC-39.8 specimen. Even though the sample containing the highest linseed oil content shows relaxation at the lowest temperature, the relationship between the oil content and *T*_g_ value can no longer be observed. Increasing the weathering time to 500 h results in further growth of the glass transition temperature for all the samples, except for the PLA-LC-30.4 one. The increase of *T*_g_ values is commonly observed in the case of weathered PLA-based samples [38] and can be explained by the growth of the crystallinity of the samples. What is more, in the case of the samples containing linseed oil, the increase of *T*_g_ may result from the antiplasticization effect, that is the leaching of the plasticizer [39,51].

### 3.5. Mechanical Properties

The data on mechanical properties of the composite samples are collected in Table 4. As can be observed, the addition of oil-rich linseed cake changed the mechanical properties of the PLA-based composites. Tensile strength decreased due to the addition of the LC, which is a common behavior in the case of the lignocellulosic waste fillers characterized with hydrophilicity and low aspect ratio [21,52,53]. What is more, the oil-rich grades of linseed cake contain a considerably low amount of rigid particles, which could contribute to the composites’ strength. Moreover, the excessive oil creates a separate phase, as proven by the SEM observations, which results in the lack of the composite’s homogeneity, hence lower values of tensile strength [30].

Tensile modulus of the PLA-based samples changes due to the addition of the waste filler, but the magnitude of such changes depends on the oil content. The PLA-LC-0.9 specimen shows higher modulus values in comparison to the neat resin, which is caused by the presence of rigid lignocellulosic particles [54,55]. Along with the growing linseed oil content E values decrease, which can be explained by both the presence of the plasticizing crude oil and a lowering content of lignocellulose [50]. Even though the oil-rich samples can be characterized by higher crystallinity in comparison to the pure PLA, as it was described earlier in this paper, the plasticizing effect of linseed oil is strong enough to suppress the stiffening caused by changes in crystallinity [56].

Elongation at break of pure, unaged polylactide was determined as 8%, which is consistent with the literature data [4]. The addition of 10 wt % of the lignocellulose-rich, particle-shaped filler resulted in the reduction of ε values. This result is common in the case of utilizing waste fillers and has been attributed to numerous factors such as lack of compatibility between the filler and the matrix, stress concentration on the lignocellulosic particles, or the presence of voids. The linseed cake-filled composites show a similar behavior but only if the crude oil content in the filler does not exceed 17.7 wt %; elongation at break noticeably increases past this point. The PLA-LC-39.8 sample reveals the ε value of 45%, which is over five times higher than in the case of pure PLA. This increase in the samples’ deformability under the static load is perhaps the most notable evidence of the plasticizing effect of linseed oil.

Unmodified, amorphous PLA is a brittle material, so the value of tensile strength around 8.5 kJ/m^2^ is a predictable result. The addition of the particle-like filler characterized with low oil content results in further deterioration of this characteristic. Similar results have been described in the case of different particle-shaped natural and waste fillers [55,57]—because of low affinity between the filler and the matrix less energy is required to cause the fracture. However, along with the growing content of crude oil within the filler, the impact strength of the samples increases. This behavior may be explained by two phenomena: The presence of linseed oil characterized by low viscosity increases the efficiency of melt blending, which allows to obtain better dispersion of the filler in the polymeric matrix [58] and it plasticizes the polymer. Therefore, more energy is consumed during the impact. What is more, linseed oil-rich samples have a higher degree of crystallinity, which can also improve their impact strength. It should also be noticed that in the case of impact strength, the influence of linseed oil is less pronounced in comparison to the elongation at break. This behavior can be explained with the fact that LC-filled composites have a non-homogeneous structure, where any discontinuities can facilitate cracks.

**Figure 7:**
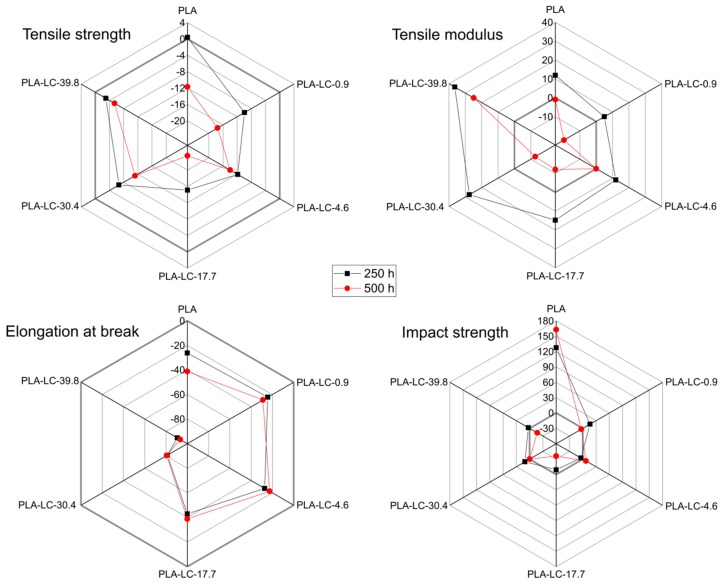
Relative change (in %) of the tensile strength, tensile modulus, elongation at break, and impact strength of the composite samples weathered for 250 and 500 h.

The relative change in the mechanical properties due to accelerated weathering is shown in Figure 7. Apart from the neat PLA, all the samples weathered for 250 h show lower values of tensile strength in comparison with the reference specimens. This result can be explained by the presence of microcracks around the filler particles (as revealed by microscopic observations), which caused a decrease in the effective cross-section of the sample. Consequently, a lower value of stress caused the fracture. The decrease in tensile strength is even more notable in the case of the composite and neat polymer samples weathered for 500 h. It may be concluded that in this case, the reasons are more complex: It is not only the presence of cracks, but also structural changes in the PLA matrix. As the DSC have shown, during accelerated weathering for 500 h both the amorphous and crystalline phase of the polymer are subjected to hydrolysis, which causes the deterioration of tensile strength.

An increase in tensile modulus can be observed in the case of the samples subjected to accelerated weathering for 250 h. This result may seem counterintuitive, but it can be explained with two phenomena. First, the weathered samples reveal higher crystallinity than the reference samples; it has been proven that the crystalline PLA is characterized with higher stiffness than the amorphous one [59]. What is more, the composite samples filled with LC can be characterized with lower values of tensile modulus because of the plasticizing effect of linseed oil. Presumably, in the case of the weathered samples, this phenomenon no longer occurred. The viscosity of linseed oil decreases at elevated temperatures, therefore it may be supposed that it leaches out of the composite during weathering through small cracks and discontinuities. Linseed oil cannot plasticize the polylactide when it is not present in the composite. Further weathering up to 500 h changes the samples tensile modulus in a different way. For all the specimens, except for the PLA-LC-39.8 one, the *E* value is equal or lower in comparison with the non-weathered samples. This phenomenon is probably caused by the already mentioned hydrolysis of the crystalline regions. The composite containing the highest amount of oil does not show this behavior—its initial low tensile modulus value was caused by the presence of the plasticizing linseed oil. Its leaching combined with the PLA solid-state structure rearrangement during weathering reduces this modification effect. Even though the hydrolysis of the polymer takes place, the stiffness is still higher than in the case of the plasticized composite.

The elongation at break values of all the samples decrease due to accelerated weathering; the higher the oil content in the filler, the more profound the decrease. The ε value measured for the PLA-LC-39.8 weathered for 250 h is almost 100% lower than in the case of the newly-manufactured sample. The decrease in the elongation at break of the weathered samples can be explained with the presence of microcracks, which make the propagation of a crack easier. However, in the case of the oil-rich specimens the effect is even more profound due to the reduction of the plasticizing effect of linseed oil, as described in the case of the tensile modulus. The samples weathered for 500 h reveal only slightly lower elongation at break values in comparison with those subjected to weathering for 250 h. It can be concluded that the relationship of the weathering time and elongation at break reduction is not linear.

The impact strength of the composite samples does not change notably after accelerated weathering, except for the neat polymer sample. In this case, a profound increase in the impact strength can be noticed. The longer the weathering time, the higher the impact strength. Similar effects have been described in various studies [38,48], which are usually explained by the modification of the interlamellar region of the polymer due to the elevated temperature, hydrolysis, and UV radiation. In the case of the composite samples this effect is suppressed by the microcracks resulting from cyclic dilatation of the phases during weathering. The two processes balance each other, therefore the overall impact strength of the LC-filled composites does not increase nor decrease.

Brittleness is a commonly used term in materials description, which was first defined by Brostow et al. as an inverse ductility defined in static and dynamic conditions. Its value B can be calculated using the following formula (5) [60]:

(5) $$B=\frac{1}{\varepsilon_{B}E{}^{\prime }}$$

where: *ε_B_*—elongation at break, *E*′—storage modulus of a specimen. In order to evaluate the brittleness of the studied samples, *G*′ values were used instead of *E*′ due to the applied DMTA analysis operated in the torsion mode.

Toughness τ of a material is the amount of energy required to crack a material. Even though it can be measured in different conditions, it is often defined according to the following formula (6) as the integrated stress–strain curve [61]:

(6) $$\tau =\int_{0}^{\varepsilon_{B}}\sigma d\varepsilon$$

As described by Brostow et al. there is an empirical correlation of brittleness and toughness [61]. For commonly-used single phase materials, it can be described using the following formula (7):

(7) $$B=\frac{b+cB}{1+aB}$$

**Figure 8:**
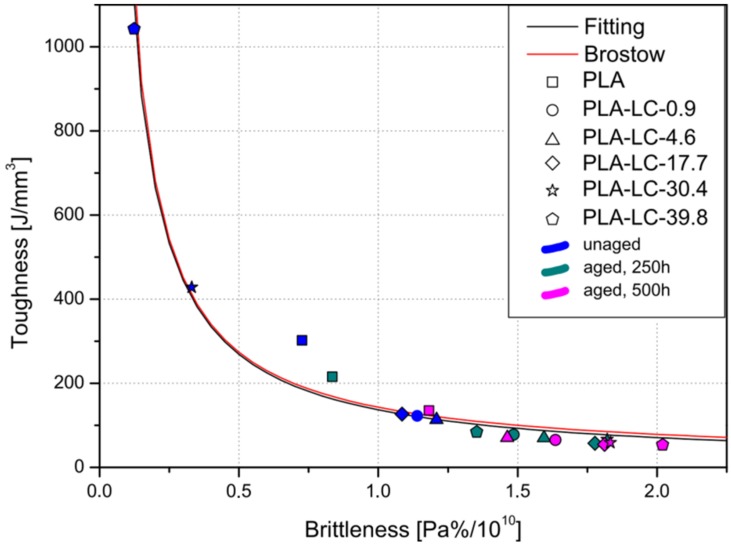
Experimental points and theoretical curves representing the correlation between the toughness and brittleness of the samples.

The coefficients a, b, c proposed by Brostow and calculated for the obtained data are presented in Table 5. The experimental points, fitted curve, and Brostow’s curve are presented in Figure 8. Even though a, b, and c coefficients obtained by Brostow and proposed in this research differ, the two curves almost overlap. The slight difference between them probably results from the fact that in the original study storage modulus used to calculate brittleness was measured in the tension mode. Nevertheless, it can be concluded that the composite materials reveal predictable brittleness–toughness relationship, which does not change due to weathering. As the original curve was plotted for single phase materials, the shape of the obtained function may also suggest good affinity between the phases in the composite.

Even though the shape of the curve does not change due to weathering, the placement of the individual points differs. It may be observed that the unaged composites, especially the ones containing higher amounts of linseed oil, can be characterized with low brittleness and high toughness. Weathering of the samples for 250 and 500 h causes a shift of the points’ positions towards lower τ values accompanied with high brittleness. In their case, the correlation between the point’s placement and oil content cannot be seen. This fact indicates that ductility of the materials is profoundly reduced during accelerated weathering of the material.

## 4. Conclusions

In the study we analyzed the influence of the linseed oil content on accelerated weathering of linseed cake-filled polylactide composites. Based on the results it can be concluded that the composite samples subjected to elevated temperature, humidity, and UV radiation undergo notable changes in their structure and properties. Initially, mutual dilatation of the waste filler and the matrix occurs. Due to different thermal expansion coefficients and mechanical properties of both phases, debonding of the linseed cake particles occurs and microcracks emerge on the surface and throughout the volume of the specimens. Excessive amounts of linseed oil contained by the linseed cake, which is not fully miscible with the polymeric matrix, leaches out of the composite using these crevices. This results in a noticeable increase in their brittleness. Simultaneously the hydrolytic degradation of the amorphous phase of the semicrystalline polymer occurs. The oil-rich samples, which are initially characterized by higher crystallinity due to the nucleating effect of LC, are therefore less susceptible to this process. However, the hydrolysis of the amorphous part combined with chemi-crystallization, caused by UV irradiation and cyclic exceeding the glass transition temperature of the material in humid conditions, results in a prominent increase in the composites’ crystallinity, hence their higher stiffness, increased glass transition temperature, and worse damping behavior. In the next stage of accelerated weathering (500 h weathering time) hydrolytic degradation of the crystalline domains begins. What is more, water penetrates the whole volume of the samples through cracks and voids resulting from debonding of the filler and leaching of the oil. This behavior is especially noticeable in the case of the oil-rich samples due to their non-homogeneous morphology. Even though the samples weathered for 500 h can be characterized with higher crystallinity than the untreated ones, their mechanical properties are visibly worse.

The addition of the linseed cake, an oil-rich waste filler, improves the properties of the polylactide-based properties such as crystallinity, elongation at break or impact strength. It can also act as a plasticizer. During weathering over a prolonged time, the presence of this filler accelerates the degradation due to the non-homogeneous structure of the composites. It can be stated that linseed-cake filled biodegradable composites are a good material for producing consumer products characterized with a short lifespan.

## Figures and Tables

**Table 1:** Measured and theoretical density and volume void content of the samples.

Sample		Measured Density	Theoretical Density	Volume Void Content
		(g/cm^3^)		(%)
Fillers	**LC-0.9**	1.4540 ± 0.00098	-	-
	LC-4.6	1.4012 ± 0.00120	-	-
	LC-17.7	1.3358 ± 0.00079	-	-
	LC-30.4	1.2732 ± 0.00098	-	-
	LC-39.8	1.1238 ± 0.00096	-	-
Unaged	PLA	1.2388 ± 0.01145	1.2400-	0.10
	PLA-LC-0.9	1.2455 ± 0.00834	1.2574	0.94
	PLA-LC-4.6	1.2488 ± 0.00800	1.2533	0.36
	PLA-LC-17.7	1.2382 ± 0.01033	1.2479	0.77
	PLA-LC-30.4	1.2276 ± 0.01105	1.2421	1.17
	PLA-LC-39.8	1.2090 ± 0.00714	1.2297	1.69
Aged, 250 h	PLA	1.2164 ± 0.00630	-	-
	PLA-LC-0.9	1.2315 ± 0.00765	-	-
	PLA-LC-4.6	1.2213 ± 0.00642	-	-
	PLA-LC-17.7	1.2154 ± 0.00704	-	-
	PLA-LC-30.4	1.2177 ± 0.00612	-	-
	PLA-LC-39.8	1.2125 ± 0.00359	-	-
Aged, 500 h	PLA	1.2234 ± 0.01043	-	-
	PLA-LC-0.9	1.2385 ± 0.00821	-	-
	PLA-LC-4.6	1.2315 ± 0.00692	-	-
	PLA-LC-17.7	1.2277 ± 0.00710	-	-
	PLA-LC-30.4	1.2173 ± 0.00599	-	-
	PLA-LC-39.8	1.2187 ± 0.00649	-	-

**Table 2:** Crystallinity (X_C_) of PLA and PLA-based composites before and after the accelerated weathering process.

Sample	XC, 1st Heating (%)			XC, 2nd Heating (%)		
	0 h	250 h	500 h	0 h	250 h	500 h
PLA	27.36	60.72 (+122%) ^a^	53.20 (−12%) ^b^	32.47	41.44 (+28%) ^a^	36.89 (+11%) ^b^
PLA-LC-0.9	27.01	71.55 (+165%) ^a^	60.35 (−16%) ^b^	40.25	44.55 (+11%) ^a^	42.32 (+5%) ^b^
PLA-LC-4.6	35.06	69.75 (+99%) ^a^	67.14 (−4%) ^b^	40.95	49.58 (+21%) ^a^	44.12 (−11%) ^b^
PLA-LC-17.7	39.49	63.46 (+61%) ^a^	57.93 (−9%) ^b^	43.70	52.45 (+20%) ^a^	42.33 (−19%) ^b^
PLA-LC-30.4	39.75	65.95 (66%) ^a^	64.54 (−2%) ^b^	57.57	58.34 (+1%) ^a^	57.40 (−2%) ^b^
PLA-LC-39.8	44.63	67.66 (+52%) ^a^	62.96 (−7%) ^b^	65.16	66.39 (+2%) ^a^	61.88 (−7%) ^b^

^a^ change of the X_C_ value in reference to the unaged sample; ^b^ change of the X_C_ value in reference to the sample aged for 250 h.

**Table 3:** Glass transition temperatures (understood as tanδ maximum) for pure PLA and composite samples weathered for different periods of time.

Sample	Untreated	Weathered for 250 h	Weathered for 500 h
	Glass Transition Temperature (°C)		
PLA	70.2	77.3	77.6
PLA-LC-0.9	69.6	76.1	77.0
PLA-LC-4.6	68.2	76.7	78.5
PLA-LC-17.7	68.2	76.3	78.5
PLA-LC-30.4	67.8	76.1	75.7
PLA-LC-39.8	67.4	75.2	77.3

**Table 4:** Mechanical properties of PLA-LC composites.

Sample	Tensile Strength (MPa)	Tensile Modulus (MPa)	Elongation at Break (%)	Impact Strength (kJ/m^2^)
PLA	74.3 ± 0.39	2270 ± 400	8.0 ± 1.8	8.39 ± 2.007
PLA-LC-0.9	59.4 ± 0.18	2430 ± 65	4.5 ± 0.2	4.44 ± 1.231
PLA-LC-4.6	56.5 ± 2.43	2270 ± 88	4.4 ± 0.4	5.17 ± 0.781
PLA-LC-17.7	53.7 ± 0.64	2160 ± 102	4.9 ± 0.3	5.66 ± 1.020
PLA-LC-30.4	46.4 ± 0.87	1890 ± 44	16 ± 6.0	5.54 ± 0.634
PLA-LC-39.8	36.7 ± 0.22	1650 ± 90	45 ± 5.4	6.85 ± 0.974

**Table 5:** Fitting coefficients proposed by Brostow and calculated for the obtained data.

Curve	a	b	c	R^2^
Brostow’s	−111	−14.102	−1640	0.934
Fitting	−6.919 × 10^8^	−9.547 ×·10^10^	−1.633·×·10^8^	0.977
